# A study indicates an essential link between a mild deterioration in excretory kidney function and the risk of neutropenia during cancer chemotherapy

**DOI:** 10.1007/s00520-023-08015-8

**Published:** 2023-09-01

**Authors:** Adriana Stryczyńska-Mirocha, Stanisław Łącki-Zynzeling, Maciej Borówka, Zofia I. Niemir, Sylwia Kozak, Aleksander J. Owczarek, Jerzy Chudek

**Affiliations:** 1 Oncology Department – Section D, Katowice Oncology Center, Raciborska 27, 40-074 Katowice, Poland; 2 Department of Internal Medicine and Oncological Chemotherapy, Medical University of Silesia, Reymonta 8, 40-029 Katowice, Poland; 3 Department of Nephrology, Transplantology and Internal Diseases, Poznań University of Medical Sciences, Poznań, Al. Przybyszewskiego 49, 60-355 Katowice, Poland; 4 Health Promotion and Obesity Management Unit, Department of Pathophysiology, Medical University of Silesia, Medyków 18, 40-752 Katowice, Poland

**Keywords:** Neutropenia, Neutropenic fever, Chemotherapy, Kidney function impairment, eGFR, CKD-EPI

## Abstract

**Purpose:**

Neutropenia, defined as a number of neutrophils in patients’ blood specimen lower than 1500 cells/μm^3^, is a common adverse event during myelosuppressive oncological chemotherapy, predisposing to febrile neutropenia (FN). Patients with coexisting moderate-to-severe chronic kidney disease (CKD) have an increased risk of FN, included in the guidelines for the primary prophylaxis of FN. However, this does not include mild kidney function impairment with estimated glomerular filtration rate (eGFR) 60–89 ml/min/1.73 m^2^. This prospective study analyzed the risk of neutropenia in patients on chemotherapy without indication for the primary prophylaxis of FN.

**Methods:**

The study enrolled 38 patients starting chemotherapy, including 26 (68.4%) patients aged 65 years or more. The median duration of follow-up was 76 days. The methodology of creatinine assessment enabled the use of the recommended CKD-EPI formula for identifying patients with a mild reduction of glomerular filtration.

**Results:**

Sixteen (42.1%) patients developed at least G2 neutropenia without episodes of FN. Only five (13.1%) patients had eGFR < 60 ml/min/1.73 m^2^, while 15 (62.5%) eGFR < 90 ml/min/1.73 m^2^. The relative risk of neutropenia in patients with impaired eGFR was over six times higher than in patients with eGFR > 90 ml/min/1.73 m^2^ (RR = 6.08; 95%CI:1.45–27.29; *p* < 0.01).

**Conclusions:**

Our observation indicates that even a mild reduction in eGFR is a risk factor for the development of neutropenia and a potential risk factor for FN.

**Supplementary Information:**

The online version contains supplementary material available at 10.1007/s00520-023-08015-8.

## Introduction

Neutropenia refers to a decreased number of circulating neutrophils in peripheral blood. Under such a condition, absolute neutrophil count (ANC) lowers below 1500 cells/μm^3^ [1]. There are several causes of neutropenia, including genetics and autoimmune diseases. However, the most common form of this complication is drug-induced [1]. The estimated incidence of drug-induced neutropenia in the general population in Europe is between 1.6 and 9.2 cases per million/per year. In the USA, it is between 2.4 and 15.4 cases per million/per year [2]. Chemotherapy-induced neutropenia (CIN) appears to be of great clinical significance, particularly in oncological patients. This complication commonly leads to febrile neutropenia (FN) in this group of patients. The latter condition impacts the dosage of chemotherapeutics or the choice of treatment schedule [3]. In Common Terminology Criteria for Adverse Events (CTCAE), FN comprises a disorder characterized by an ANC lower than 1000/μm^3^ and a single rise in temperature above 38.3 °C (101 °F) or a sustained temperature ≥ 38 °C (100.4 °F) for more than 1 h [4]. In the USA, FN occurs in 7.83 cases per 1000 subjects treated for cancer [5]. The mortality rate in patients with solid tumors that developed FN ranges from 2.6 to 7.0% [5].

Furthermore, FN-related hospitalizations generate high costs to health systems [6]. The above facts resulted in the publishing by the European Society for Medical Oncology [7] and the American Society of Clinical Oncology [8] guidelines for FN prophylaxis. Both societies recommend the use of granulocyte colony-stimulating factor (G-CSF).

Some risk factors for neutropenia help assess the probability of lowered ANC occurrence and thus select the appropriate prophylactic strategy. Patients of older age with poor performance status, low body mass index (BMI), low body surface area (BSA), low baseline lymphocyte or neutrophil count, and suffering from cardiovascular and kidney diseases are at risk of neutropenia. In addition, the type and advancement of malignancy and the presence of specific genetic mutations might influence the risk of the development of FN. Finally, treatment with some chemotherapeutic agents increases the risk of FN [9]. Patients with acute kidney injury (AKI) and chronic kidney disease (CKD) are more prone to FN than patients without kidney disease [10]. Primary prophylaxis with G-CSF reduces the risk of FN in patients with solid tumors [11–13]. Current recommendations encourage the use of G-CSF in the primary prevention of FN in patients with CKD and the estimated glomerular filtration rate (eGFR) < 60 ml/min/1.73 m^2^ [7, 8].

The relationships between cancer treatment strategies and kidney functions are very complex. CKD might increase the risk of numerous cancers [14], and conversely, malignancies might lead to kidney disorders [15]. In addition, oncologic patients with CKD have a poorer survival prognosis [16]. Renal function in cancer patients is also crucial in anti-cancer treatment due to the renal elimination of numerous xenobiotics. Chemotherapeutic agents are themselves commonly nephrotoxic. On the other hand, they are causing cancer cell destruction, resulting in the release of substances that may further impair renal function [17]. Thus, chemotherapeutic doses in cancer patients must be thoroughly adjusted to patients’ renal function [17].

One of the most popular methods of kidney function assessment is estimating the glomerular filtration rate (eGFR) based on serum creatinine, sex, age, and race, using available formulas [18]. Equations such as aMDRD (abbreviated Modification of Diet in Renal Disease Study), CKD-EPI (Chronic Kidney Disease Epidemiology Collaboration), and Cockcroft-Gault formulas are commonly used in oncological patients. Each of them has some advantages and limitations [19]. Albeit, there is accumulating evidence about the significant advantages of the CKD-EPI formula [20], notably in cancer patients [19], which is in line with nephrological recommendations [21]. The aMDRD equation is still commonly used by some laboratories assessing serum creatinine with the old Jaffa method [22]. The CKD-EPI formula is exceptionally accurate for eGFR higher than 60 mL/min/1.73 m^2^. All the strategies mentioned above enable a reliable assessment of mildly impaired kidney function compared with the results of serum creatinine measurement by standardized isotope dilution mass spectrometry (IDMS) [23].

In this study, we aimed to assess whether mildly decreased eGFR (60–89 ml/min/1.73m^2^, assessed with CKD-EPI formula) increases the risk of neutropenia during cancer chemotherapy, where primary prophylaxis of FN with G-CSF is not justified.

## Materials and methods

The Bioethics Committee of the Medical University of Silesia in Katowice (approval no. KNW/0022/KB1/24/19) approved the study. All subjects signed informed consent for participating in the study conducted in the Oncology Department D of the Cancer Center in Katowice from March 2019 to April 2020.

The inclusion criteria were initiation of myelosuppressive adjuvant or palliative chemotherapy (CTH) in patients with solid cancers without a high risk of FN as defined by the current international guidelines [24]. There were no exclusion criteria except indications for primary prophylaxis with G-CSF and co-administration of glucocorticoids and potentially myelosuppressive drugs except prescribed during CTH schedule.

### Data collection

Demographic (sex, age), anthropometric (weight, body mass index — BMI, body surface area — BSA), clinical (occurrence of diabetes, cardiovascular disease: hypertension, coronary artery disease), and biochemical data (serum creatinine, total blood count, urine analysis) were analyzed. BMI was calculated with the standard formula and expressed in kg/m^2^. BSA was estimated using the formula of DeBois [25]. The CKD-EPI equation was applied to calculate eGFR [20].

$$\textrm{GFR}=141\times \min {\left(\textrm{Scr}/\upkappa, 1\right)}^{\upalpha}\times \max {\left(\textrm{Scr}/\upkappa, 1\right)}^{-1.209}\times {0.993}^{\textrm{Age}}\times 1.018\left[\textrm{if}\ \textrm{female}\right]$$

where Scr, serum creatinine; *κ*, 0.7 for females and 0.9 for males; *α*, − 0.329 for females and − 0.411 for males; min, minimum of Scr/κ or 1; max, maximum of Scr/*κ* or 1.

### Study endpoints

The main study endpoint was the occurrence of neutropenia (at least grade 2) or FN during the CTH conducted as planned or until termination due to unacceptable toxicity or disease progression or eventual initiation of therapy with G-CSF. Grade 2 neutropenia was defined, according to the Common Terminology Criteria for Adverse Events (CTCAE) v5.0, as neutrophil count decreased < 1500–1000/μL (< 1.5 − 1.0 × 10^9/L) [4].

The secondary end-points were occurrence of other symptoms of myelosuppression: at least grade 2 thrombocytopenia < 75,000/ μL (< 75 × 10^9 /L) and grade 2 anemia < 10 g/dL [4].

### Statistical analysis

The STATISTICA 13.1 (TIBCO Software Inc., Palo Alto, CA, USA) and STATA 13.1 (StataCorp, Lakeway Drive, Texas, USA) served for data analysis. A *p* value < 0.05 was set as the level of statistical significance with two-sided tests. No data imputation methods were used. Data with a normal distribution are presented as mean ± standard deviation and with a non-normal distribution or heavily skewed as median (lower quartile – upper quartile). Data on the nominal and interval scales are presented as counts and percentages. The Shapiro-Wilk test and the quantile-quantile (Q-Q) plot verified data distribution. The *χ*^2^ or the Fisher test for variables on the nominal and ordinal scale and the Student’s *t* test for independent variables for data on the interval scale were used for data comparison. In the case of skewed data, a logarithmic normalization was done. The analysis of variance with repeated measurements and post hoc tests (contrast analyzes) were used to analyze the longitudinal data on an interval scale. The Cox proportional hazard analysis was used to assess risk factors for neutropenia. The results were presented as the hazard ratio (HR) with a corresponding 95% confidence interval (CI) and the value of statistical significance level. In each case, the fulfilment of the proportional hazard assumption was assessed based on the Schoenfeld residuals (_PPH_). The risk factors relevant to the univariable analysis were included in the multivariable analysis. Survival analysis was based on Kaplan-Meier curves, and their comparisons were made with the log-rank test.

## Results

### Characteristics of the patients

Thirty-eight patients, aged 68 ± 6 years (range: 57–82), treated systemically for colorectal cancer (C18–C20, *N* = 34) or non-small cell lung cancer (C34, *N* = 4) without primary prevention of FN were analyzed. Treatment schedules for colorectal cancer comprised LvFu2 (*N* = 6), FOLFOX4 (*N* = 19), FOLFOX4 with bevacizumab (*N* = 2), FOLFOX4 with panitumumab (*N* = 1), FOLFIRI (*N* = 2), FOLFIRI with panitumumab (*N* = 1), FOLFIRI with bevacizumab (*N* = 1), or FOLFIRI with aflibercept (*N* = 2). The therapy of non-small cell lung cancer included cisplatin with vinorelbine (*N* = 1) or pemetrexed (*N* = 3). The cohort included 14 (36.8%) women and 26 (68.4%) subjects at least 65 years old. Eighteen (47.4%) patients had a diagnosis of cardiovascular disease. The most common were hypertension (*N* = 15; 39.5%) and coronary artery disease (*N* = 6; 15.8%). Moreover, eight (21.0%) participants had diabetes. Before the CTH, significant weight loss was noted in 15 (39.5%) patients (Table 1).

**Table 1:** Baseline patients’ characteristics and comparison of clinical parameters between women and men

	Whole group *N* = 38	Men *N* = 24 (63.2%)	Women *N* = 14 (36.8%)	*p* value
Age [years]	68 ± 6	68 ± 6	69 ± 7	0.63
Age ≥ 65 years [*N* (%)]	26 (68.4)	16 (66.7)	10 (71.4)	0.76
Body weight [kg]	75.3 ± 17.0	81.4 ± 17.4	64.6 ± 9.5	< 0.001
Weight loss before CHT [N (%)]	15 (39.5)	9 (37.5)	6 (42.9)	0.74
Body surface [m^2^]	1.86 ± 0.23	1.95 ± 0.22	1.69 ± 0.16	< 0.001
BMI [kg/m^2^]	26.7 ± 4.6	28.0 ± 4.6	24.6 ± 4.0	< 0.05
Comorbidities				
Diabetes [*N* (%)]	8 (21.1)	7 (29.2)	1 (7.4)	0.21
Hypertension [*N* (%)]	15 (39.5)	11 (45.8)	4 (28.6)	0.33
Coronary artery disease [*N* (%)]	6 (15.8)	6 (25.0)	0	0.07
Cardiovascular disease [*N* (%)]	18 (47.4)	14 (58.3)	4 (28.6)	0.10
Baseline laboratory work-ups				
Serum creatinine [mg/dL]	0.90 ± 0.27	0.94 ± 0.25	0.84 ± 0.28	0.22
eGFR [ml/min/1.73 m^2^]	90.8 ± 28.8	97.9 ± 31.8	78.8 ± 18.2	< 0.05
eGFR < 90 [ml/min/1.73 m^2^]	22 (57.9)	12 (50.0)	10 (71.4)	0.20
Erythrocytes [10^6^/μL]	4.3 ± 0.6	4.5 ± 0.5	3.9 ± 0.5	< 0.01
Thrombocytes [10^3^/μL]	284 ± 118	276 ± 128	298 ± 100	0.58
Leukocytes [10^3^/μL]	7.6 ± 2.1	7.9 ± 2.1	7.1 ± 1.9	0.23
Neutrophils [10^3^/μL]	4.7 ± 1.8	4.9 ± 1.9	4.2 ± 1.6	0.29
Hemoglobin [g/dL]	12.5 ± 1.8	13.1 ± 1.8	11.5 ± 1.0	< 0.01
Anemia (Hb < 11 g/L)	6 (15.8)	3 (12.5)	3 (21.4)	0.65
Hb < 12 g/L	15 (39.5)	5 (20.8)	10 (71.4)	< 0.01
Thrombocytopenia [*N*(%)]	2 (5.3)	2 (8.3)	0	0.52
Follow-up (at any time)				
Neutropenia G_2-4_ [*N* (%)]	16 (42.1)	9 (37.5)	7 (50.0)	0.45
Anemia [*N* (%)]	9 (23.7)	4 (16.7)	5 (35.7)	0.24
Thrombocytopenia [*N* (%)]	7 (18.4)	6 (25.0)	1 (7.1)	0.23

*N* number of patients, *CHT* chemotherapy, *BMI* body mass index, *eGFR* estimated glomerular filtration rate, *Hb* hemoglobin

Initially, 15 (62.5%) patients had reduced eGFR (< 90 ml /min/1.73 m^2^). Values below 60 ml/min/1.73 m^2^ were found only in five (13.1%) patients.

The median duration of follow-up of the study (until the onset of neutropenia or completion of CTH) was 76 days (lower and upper quartiles 50 and 161 days, respectively). The maximum observation time was 330 days. In seven patients, the scheduled treatment was discontinued without the occurrence of neutropenia. The reasons for the unscheduled CTH termination were disease progression (*N* = 9), deterioration in physical function (*N* = 2), stroke (*N* = 1), and in one case, death. In two cases, contact with the patient was lost. There was any case of G-CSF use before the onset of at least G2 neutropenia.

Sixteen (42.1%) patients developed the primary endpoint (neutropenia G_2-4_) during the follow-up. In this group, G_2_ neutropenia occurred in three, G_3_ in nine, and G_4_ in four patients. There were no episodes of FN. The median time to onset neutropenia was 57 days (lower and upper quartiles 42 and 91 days, respectively), with a maximum time of 193 days.

Initially, hemoglobin count lower than 11 g/L, which according to NCCN guidelines should encourage assessment of anemia, occurred in six patients (15.8%). It persisted for the first two visits in four, and in further two also included the third and fourth visits. During the follow-up, at least one episode of G_2_ anemia occurred in five (13.2%) patients.

Thrombocytopenia (< 150 × 10^9 /L ) was initially found in two (5.3%) patients and any patient developed at least CTCAE grade 2 thrombocytopenia during the follow-up [4].

### Comparison of the group of women and men

Table 1 compares women and men regarding comorbidities and the baseline clinical data. Features such as lower body weight, BMI, hemoglobin, and eGFR values and smaller body surface area were more frequent in women. No other statistically significant differences between both groups were found, except for the more frequent occurrence of coronary artery disease in men (a tendency to statistical significance).

### Comparison of patients with and without neutropenia

Table 2 compares patients with and without neutropenia (at least G_2_ during CTH). Neutropenic patients were much more frequently older, had lower eGFR levels, and more often suffered from hypertension (a tendency to statistical significance). No other statistically significant differences were found.

**Table 2:** Comparison between patients with and without neutropenia (at least G_2_ during CTH)

	Without neutropenia *N* = 16 (57.9%)	Neutropenia *N* = 22 (42.1%)	*p* value
Women [*N* (%)]	7 (31.8)	7 (43.7)	0.45
Age [years]	69 ± 4	68 ± 9	0.70
Age ≥ 65 years [*N* (%)]	18 (81.8)	8 (50.0)	< 0.05
Body weight [kg]	72.7 ± 13.9	78.8 ± 20.4	0.28
Weight loss before CHT [*N* (%)]	10 (45.4)	5 (31.2)	0.51
Body surface [m^2^]	1.83 ± 0.22	1.89 ± 0.25	0.47
BMI [kg/m^2^]	23.7 ± 5.7	26.8 ± 7.5	0.16
Comorbidities			
Diabetes [*N* (%)]	5 (22.7)	3 (18.7)	1.00
Hypertension [*N* (%)]	6 (27.3)	9 (56.3)	0.07
Coronary artery disease [*N* (%)]	3 (13.6)	3 (18.7)	0.68
Cardiovascular disease [*N* (%)]	8 (36.4)	10 (62.5)	0.11
Baseline laboratory work-ups			
Serum creatinine [mg/dL]	0.82 ± 0.20	1.02 ± 0.31	< 0.05
eGFR [ml/min/1.73m^2^]	99.4 ±26.9	79.1 ± 27.9	< 0.05
eGFR < 90 [*N* (%)]	8 (36.4)	14 (87.5)	< 0.01
Anemia (Hb < 11 g/L) [*N* (%)]	5 (22.7)	1 (6.2)	0.37
Hb < 12 g/L [*N* (%)]	8 (36.4)	7 (43.7)	0.64
Thrombocytopenia [*N* (%)]	0	2 (12.5)	0.17
Follow-up (at any time)			
Anemia [*N* (%)]	5 (22.7)	4 (25.0)	1.00
Thrombocytopenia [*N* (%)]	2 (9.1)	5 (31.2)	0.11

*N* number of patients, *CHT* chemotherapy, *BMI* body mass index, *eGFR* estimated glomerular filtration rate, *Hb* hemoglobin

A significant change in the platelet counts over time was demonstrated, yet without clinical complications (*p*_*Time*_ < 0.05; *p*_*Time* × *group*_ = 0.42). There were no differences between both groups at individual observation time points. In neutropenic and non-neutropenic groups, the decrease in platelets was as follows: from baseline counts of 306 ± 100 to 225 ± 89 × 10^3^/μL (*p* < 0.05) and from 282 ± 118 to 197 ± 40 × 10^3^/μL (*p* < 0.01), respectively.

There was also a significant change over time in blood counts of neutrophils, yet without negative consequences (*p*_*Time*_ < 0.05; *p*_*Time* × *group*_ = 0.59). No differences between both groups at individual observation time points were observed. In the group with neutropenia, the number of neutrophils at the fourth measurement was significantly lower than at the baseline (2.30 ± 1.83 vs. 4.57 ± 1.58 × 10^3^/μL; *p* < 0.05).

**Fig. 1:**
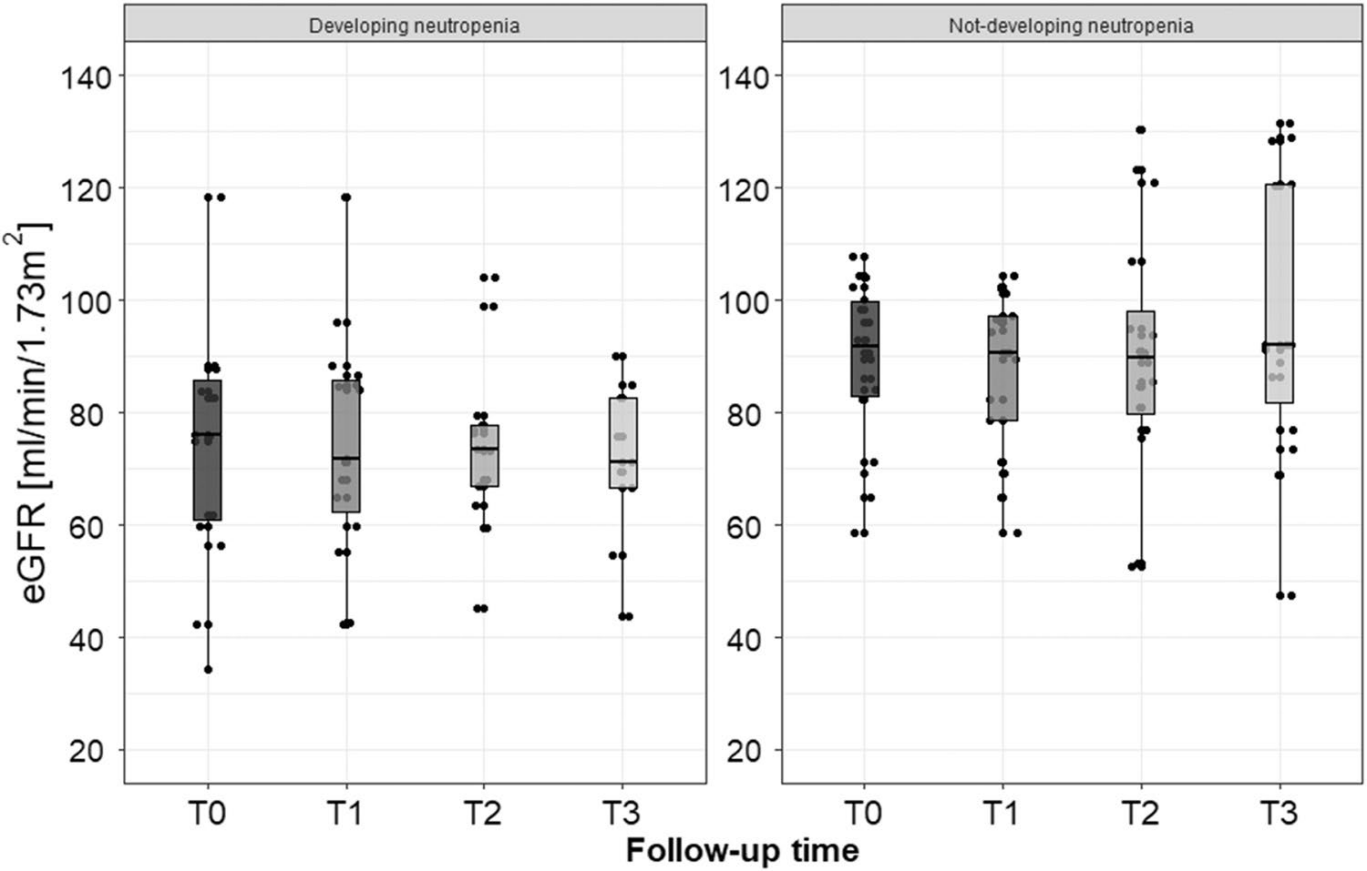
The box plot of eGFR values in patients without and with neutropenia during follow-up (before the first four CTH schedules)

There were no statistically significant differences between both groups in serum creatinine (*p*_*Time*_ = 0.81; *p*_*Time × group*_ = 0.52) and the eGFR-EKD-EPI (*p*_*Time*_ = 0.95; *p*_*Time × group*_ = 0.71). However, during the observation period, patients with neutropenia had statistically significantly lower eGFR values than patients without neutropenia (*p* < 0.05 at each time point) shown in Fig. 1.

### Risk factors for neutropenia

Table 3 presents the results of the univariable Cox proportional analysis. The critical risk factor for the occurrence of neutropenia was eGFR < 90 ml/min/1.73 m^2^ (HR = 6.5), while surprisingly, the preventive factor was age ≥ 65 (HR = 0.26). Age ≥ 65 years and decreased eGFR were included in the multivariable analysis. The direction of the influence of variables remained unchanged. The elderly subjects had a lower risk of developing neutropenia (HR = 0.27; 95% CI: 0.10–0.77; *p* < 0.05), contrary to the higher risk of this complication in patients with reduced eGFR (HR = 6.33; 95% CI: 1.39–28.77; *p* < 0.05).

**Table 3:** One-way analysis of Cox proportional hazard–risk factors for neutropenia in the studied group of patients

	HR	± 95% CI	*p* value	_PPH_ test
Women	1.60	0.59–4.34	0.35	0.62
Age [5 years]	0.77	0.48–1.23	0.28	0.83
Age ≥ 65 years	0.26	0.09–0.70	< 0.01	0.47
Body weight [1 kg]	1.02	0.99–1.06	0.22	0.23
Weight loss before CHT	0.59	0.20–1.70	0.33	0.41
Body surface [1 m^2^]	1.63	0.16–16.62	0.68	0.77
Baseline BMI [1 kg/m^2^]	1.04	0.93–1.17	0.49	0.96
Diabetes	0.80	0.23–2.81	0.72	0.78
Cardiovascular disease	1.93	0.70–5.35	0.20	0.08
Hypertension	2.06	0.76–5.56	0.16	0.12
Coronary artery disease	1.66	0.46–5.89	0.44	0.42
eGFR [10 mL/min/1.73 m^2^]	0.79	0.62–0.99	< 0.05	0.15
eGFR < 90 mL/min/1.73 m^2^	6.50	1.46–28.87	< 0.05	0.35
Hemoglobin [< 12 g/L]	1.14	0.42–3.08	0.79	0.70
Thrombocytes [10^3^/μL]	0.95	0.89–1.01	0.10	0.14
Leukocytes [10^3^/μL]	0.82	0.61–1.10	0.18	0.36
Neutrophils [10^3^/μL]	0.80	0.56–1.14	0.21	0.24

*N* number of patients, *CHT* chemotherapy, *BMI* body mass index, *eGFR* estimated glomerular filtration rate, *HR* hazard ratio, *CI* confidence interval, *PPH* Schoenfeld residuals test *p* value

*For the whole value ranges

### Survival analysis for neutropenia

**Fig. 2:**
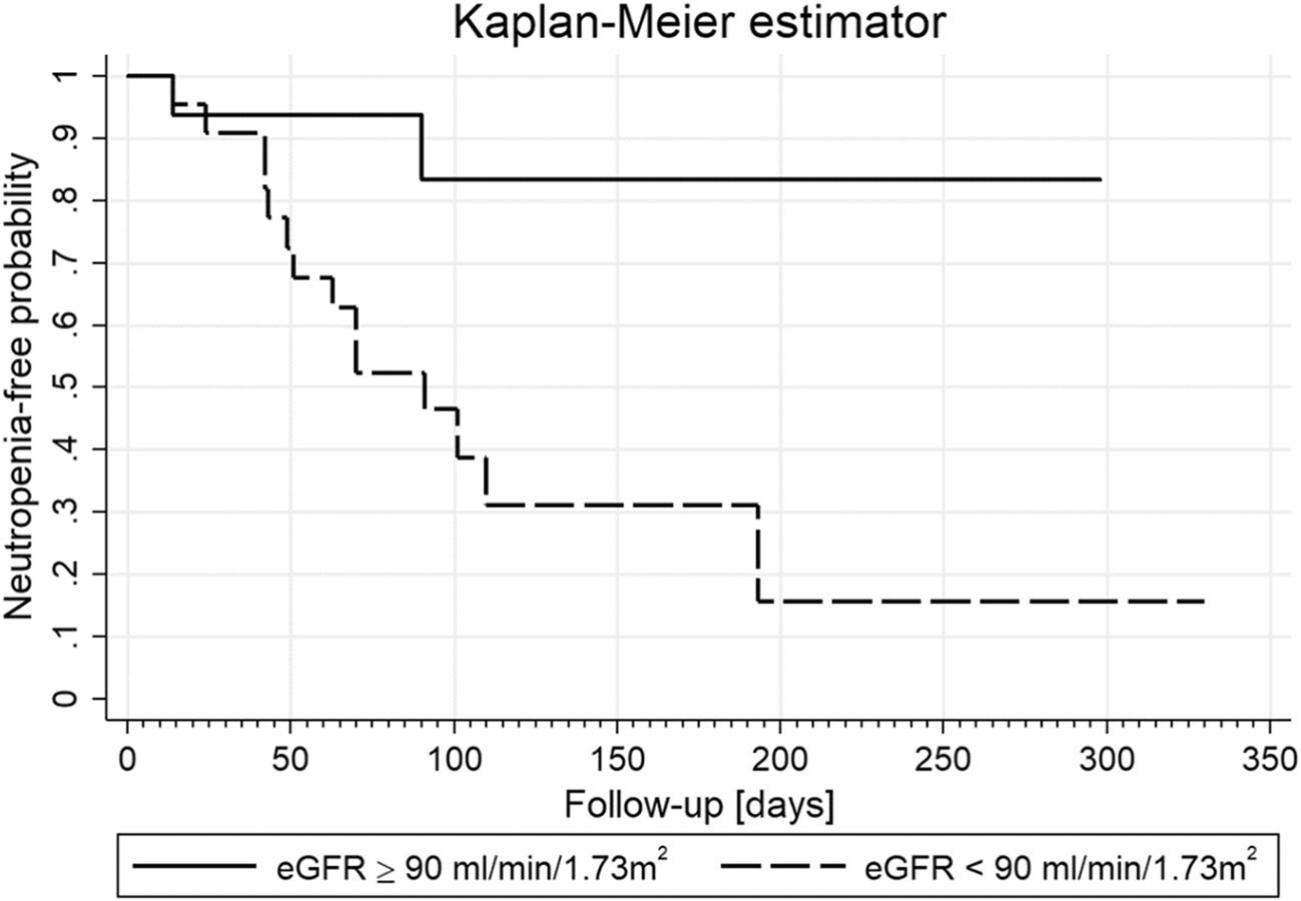
Kaplan-Meier survival curves of the probability of G_2-4_ neutropenia during the follow-up in groups of patients with and without decreased eGFR

The Kaplan-Meier curves of the probability of neutropenia occurrence in the subgroups with normal and decreased eGFR are shown in Fig. 2.

The incidence of neutropenia in the whole group was 3.99 per 1000 person-days (95% CI: 2.44–6.52/1000). In the whole group, the lower quartile and median of neutropenic-free survival days were 70 and 193, while in the neutropenic group, there were 42 and 51 days, respectively.

There was a statistically significant difference in Kaplan-Meier curves between patients with and without decreased eGFR (log-rank < 0.01). The incidence of neutropenia in the group with and without decreased eGFR was 6.97 (95% CI: 4.13–11.77) and 1.00 (95% CI: 0.25–4.00) per 1000 person-days, respectively. The relative risk of neutropenia for patients with decreased eGFR values was more than six times higher than for patients with eGFR > 90 ml/min/1.73 m^2^ (RR = 6.08; 95% CI: 1.45–27.29; *p* < 0.01). The median survival time without neutropenia in the group with decreased eGFR was 91 days; the lower and upper quartiles were 49 and 193 days, respectively. While the estimated mean survival time without neutropenia in the normal and low eGFR groups was 295 (95% CI: 207–309) and 125 (95% CI: 72–178) days, respectively.

### Comparison of patients with normal and lowered eGFR

Patients with reduced eGFR did not differ in age and occurrence of comorbidities, except close to the statistically significant tendency for a lower occurrence of coronary artery disease (*p* = 0.07). Of note, subjects with reduced eGFR had a higher incidence of neutropenia (63.4% vs. 12.5%; *p* < 0.001). No other statistically significant differences were found (shown in Supplementary Table 1).

## Discussion

The known risk factors indicated in the guidelines for the prevention of FN are age, cardiovascular diseases, poor performance status, history of prior FN, advanced disease, mucositis, no antibiotic prophylaxis, or G-CSF use. However, there are still limited studies providing data in this area considering other, particularly chronic comorbidities. Here, we present our results showing the relationship between mildly impaired kidney function with the occurrence of neutropenia in cancer patients.

In our cohort, 42.1% of patients developed G_2-4_ neutropenia during the follow-up, which was the key result of the analysis. There were no FN episodes, probably due to the relatively small size of the study groups (our study’s main drawback).

### Age and performance status

Age is one of the essential factors in the development of cancer. The peak incidence occurs at 65–75 years of age due to fewer older age groups and more frequent failure to recognize these diseases among the oldest ones. This demographical association directly translates into an increased number of patients over 65 qualified for cancer CTH [26].

Patients over 65 are heterogeneous regarding biological status, comorbidities, and organ function. These factors can be decisive in qualifying for systemic treatment with cytostatic drugs [27].

Before initiating the treatment, the patients undergo assessment according to the ECOG or the Karnowski performance rating scales. Adjuvant or palliative treatment is those with ECOG performance of 0–1 points or 70–100%, according to Karnowski’s scale. Less frequently, patients with lower performance characteristics (ECOG 2) are qualified for systemic treatment with cytostatic drugs, especially those not burdened with multiple diseases [27]. Older patients are more susceptible to treatment complications with cytostatic drugs, particularly myelotoxicity. It is directly related to the reduction of organ reserves and a lower ability to regenerate, related to age and comorbidities-related organ damage. Moreover, there are also changes in the pharmacokinetics of drugs, resulting in impaired elimination of drugs due to liver and kidney dysfunctions and drug interactions [28].

Age is one of the most frequently mentioned risk factors of FN in the medical literature [9, 29]. Surprisingly, in our study, neutropenia occurred significantly more often in patients younger than 65 years compared to older ones. These results might be due to the enrolment of elderly patients without numerous risk factors requiring primary FN prevention. Such cases were previously mentioned in the literature [30]. Moreover, in our group of patients, arterial hypertension occurred slightly more frequently in younger patients (< 65 years) than in the older group.

### Comorbidities (hypertension, coronary artery disease, diabetes)

Cardiovascular diseases (CVD) remain a significant cause of morbidity and mortality in patients with cancer [31, 32]. Among all CVD, hypertension is the most commonly reported in patients undergoing CTH [33].

We found a close to statistical significance towards the more frequent hypertension prevalence in younger neutropenia patients. There was also the additional coincidence of lower eGFR in this subgroup of patients (mainly in the range from 60 to 89.9 ml/min/1.73 m^2^). In contrast, we did not observe any difference between coronary artery disease and diabetes patients regarding neutropenia. Supporting our results, Chao et al., in a study including 19,160 patients, showed that diabetes did not increase the risk of neutropenia. However, the same study did not reveal the association between hypertension, neutropenia, and the occurrence of FN. On the other hand, the authors found a statistically significant relationship between FN and other comorbidities such as chronic obstructive pulmonary disease, congestive heart failure, human immunodeficiency virus infection, autoimmune diseases, peptic ulcer, renal disease, and thyroid disorder [34].

### Effect of chemotherapy on renal impairment

Intriguingly, patients with neutropenia had statistically significantly lower eGFR values than patients without neutropenia. In addition, it considered the entire follow-up. Furthermore, the demonstrated relationship was due to impaired renal function before introducing the therapy. Nevertheless, during the therapy, the excretory function of the kidneys was stable in both subgroups.

### eGFR 60-89 as a potential risk factor for FN

There was a statistically significant difference in the incidence of neutropenia (Kaplan-Meier curves) between the patients without and with reduced eGFR. This observation seems to be very important clinically. CKD with eGFR < 60 ml/min/1.73m^2^ is a known risk factor in the primary prevention of FN [23]. However, we did not find a study that evaluated whether a slight impairment in renal function with eGFR < 90 ml/min/1.73 m^2^ is a risk factor for FN. At least partly responsible for such results may be the earlier determination of serum creatinine with the Jaffe method and consecutive estimation of eGFR based on the commonly used aMDRD formula in laboratories until recently. The introduction of a new method for the determination of serum creatinine concentration with the enzymatic method enabled the use of the CKD-EPI formula and improved eGFR estimation, especially in the range from 60 to 89.9 ml/min/1.73 m^2^ [23]. Since 2021, this method is used for the calculation of eGFR in cancer patients in North America [21].

Our study implies that proper kidney function is essential for lower toxicity of cancer therapy. Even a slight impairment in excretory kidney function increases cytostatic therapy’s risk of systemic myelotoxicity. Nevertheless, our results have a pilot character requiring verification performed on a larger group of patients.

To some extent, as justification for our results, we can blame the pandemic of COVID-19. Unfortunately, our patient recruitment occurred during the early pandemic period of COVID-19. Hence, this resulted in lower participation of patients with ECOG 2 performance. During the pandemic, according to the ESMO recommendations, cytostatic treatment could be applied to non-disabled patients with the allowance of G-CSF primary prevention in patients with multiple risk factors for developing neutropenia during CTH regimens with intermediate risk of FN [35].

## Conclusions

Reduction in the glomerular filtration rate below 90 ml/min/1.73m^2^ is a significant risk factor for developing neutropenia during cancer chemotherapy. Whether it may result in more frequent episodes of FN requires further investigation.

## Supplementary information

ESM 1

## Data Availability

The data presented in this study are available on request from the corresponding author.
