# Supplementary figures and images for: A study indicates an essential link between a mild deterioration in excretory kidney function and the risk of neutropenia during cancer chemotherapy
Source: Support Care Cancer. 2023 Sep 1;31(9):549. doi: 10.1007/s00520-023-08015-8 (PMC10473980; doi:10.1007/s00520-023-08015-8)

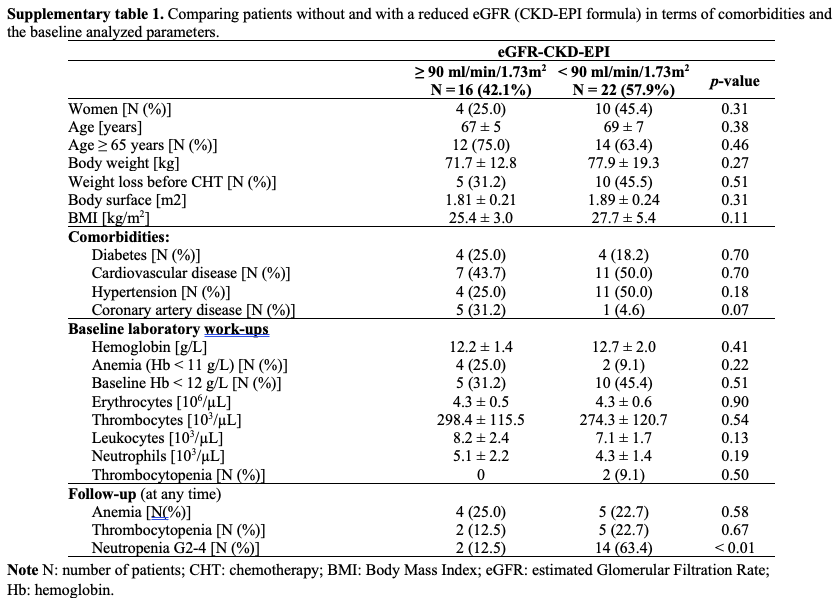

Supplement: Supplementary file 1 — ESM 1 [file 520_2023_8015_MOESM1_ESM.png]
